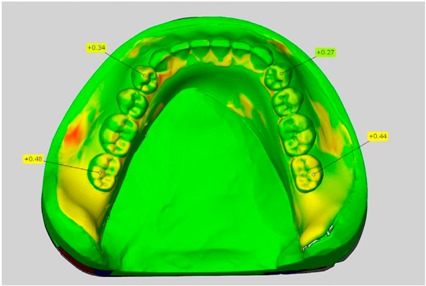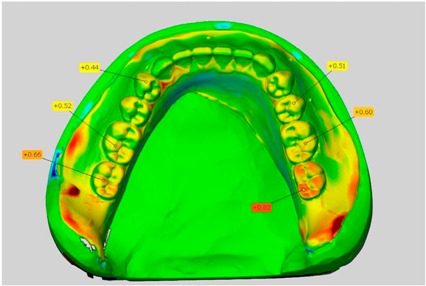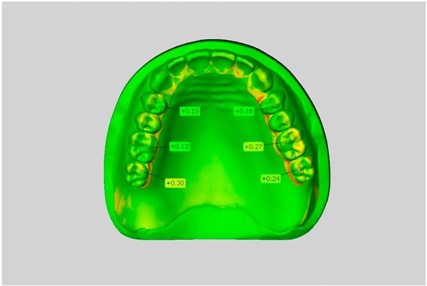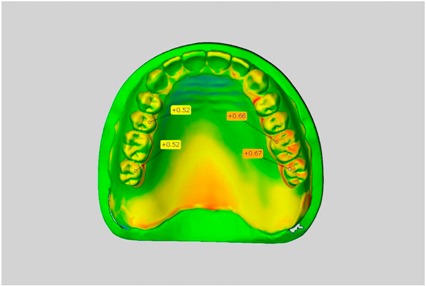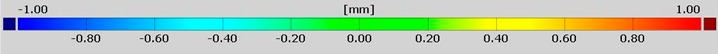# Accuracy of Digitally Fabricated Wax Denture Bases and Conventional Completed Complete Dentures

**DOI:** 10.3390/dj5040036

**Published:** 2017-12-19

**Authors:** Bogna Stawarczyk, Nina Lümkemann, Marlis Eichberger, Timea Wimmer

**Affiliations:** Department of Prosthetic Dentistry, University Hospital, LMU Munich, Goethestrasse 70, 80336 Munich, Germany; nina.luemkemann@med.uni-muenchen.de (N.L.); marlis.eichberger@med.uni-muenchen.de (M.E.); timea.wimmer@med.uni-muenchen.de (T.W.)

**Keywords:** complete denture, CAD/CAM, accuracy, Ceramill Full Denture Workflow

## Abstract

The purpose of this investigation was to analyze the accuracy of digitally fabricated wax trial dentures and conventionally finalized complete dentures in comparison to a surface tessellation language (STL)-dataset. A generated data set for the denture bases and the tooth sockets was used, converted into STL-format, and saved as reference. Five mandibular and 5 maxillary denture bases were milled from wax blanks and denture teeth were waxed into their tooth sockets. Each complete denture was checked on fit, waxed onto the dental cast, and digitized using an optical laboratory scanning device. The complete dentures were completed conventionally using the injection method, finished, and scanned. The resulting STL-datasets were exported into the three-dimensional (3D) software GOM Inspect. Each of the 5 mandibular and 5 maxillary complete dentures was aligned with the STL- and the wax trial denture dataset. Alignment was performed based on a best-fit algorithm. A three-dimensional analysis of the spatial divergences in *x*-, *y*- and *z*-axes was performed by the 3D software and visualized in a color-coded illustration. The mean positive and negative deviations between the datasets were calculated automatically. In a direct comparison between maxillary wax trial dentures and complete dentures, complete dentures showed higher deviations from the STL-dataset than the wax trial dentures. The deviations occurred in the area of the teeth as well as in the distal area of the denture bases. In contrast, the highest deviations in both the mandibular wax trial dentures and the mandibular complete dentures were observed in the distal area. The complete dentures showed higher deviations on the occlusal surfaces of the teeth compared to the wax dentures. Computer-aided design/computer-aided manufacturing (CAD/CAM)-fabricated wax dentures exhibited fewer deviations from the STL-reference than the complete dentures. The deviations were significantly greater in the vicinity of the denture teeth area and the bases. The conventional transfer of CAD/CAM-fabricated wax dentures into acrylic resin leads to the highest deviations from the STL-reference.

## 1. Introduction

Lately, the application of computer-aided design/computer-aided manufacturing (CAD/CAM) technology in the construction and manufacturing process of complete dentures has increased considerably. The fabrication of complete dentures using a CAD/CAM system has the potential to simplify the fabrication process. In general, the production of complete dentures is known to be a complex and time-consuming procedure, in which precision is decisive. The option to fabricate complete dentures by means of the CAD/CAM technology provides both a high utilization of laboratory CAD/CAM systems—and thus efficiency—and also high process reliability. For this reason, several manufacturers have developed different approaches for digitizing this process over the past few years. Now, the operator is able to create reproducible data, but whether the subsequent digital fabrication steps provide sufficient accuracy remains to be proved. Many clinical case reports have already been published on this subject [1,2,3,4,5,6]. In vitro studies on the accuracy of digitally fabricated complete dentures are scarce. In one of the first investigations concerning the accuracy of complete dentures, a complete titanium denture base plate was fabricated by a CAD/CAM/laser rapid forming (LRF) system [7]. To evaluate the fit of the plate, a virtual adaptation test was carried out. The test measured the profiles of the laser free-formed denture plate, as well as those of the edentulous dental cast. The values were compared, and a mean deviation of 0.34 mm was observed [7]. In a further study, a complete denture set, which was worn by a patient, was reformed in the dental practice, and three-dimensional (3D) cone beam computed tomography (CBCT) scans were performed. The obtained 3D data were solely used for the mucosal surface. With the CAD software, the morphological data for the new dentures were fabricated and the denture teeth were cut out. The new complete denture base was fabricated from an acrylic resin block by a CNC (computerized numerical control) machining center. Finally, the artificial teeth selected on the CAD application were bonded to the denture bases. Then, the dentures were digitized with a 3D digitizer, and the deviations from the 3D master complete denture were measured. For the occlusal surface, the average deviations amounted to 0.50 mm [8]. Another study described a clinical impression method for obtaining denture base morphology, as well as the phonetic and muscular locations of the artificial denture teeth. Afterwards, the recorded information was scanned, and the bases of the complete denture were designed virtually, and then milled from resin [9].

Six years later, an in vitro study was conducted to assess the influence of different palatal forms (shallow and the deep) on the displacement of the denture teeth during the processing of complete dentures [10]. To this end, 10 maxillary casts were fabricated by means of duplication for each palatal form. Base plates were made, and denture teeth were set in their anatomic positions. Metal pins served as reference points, and were placed on the first molars and in the incisive papilla area. Casts were scanned using i-CAT cone beam 3D dental imaging system. The distances between the apices of the metallic pins and the fitted reference points were measured in buccopalatal axes at 3 stages (wax-up, deflasking and after finishing). During all processing stages, and for both palatal forms, the results showed statistically significant movements of teeth [10].

The dimensional changes that appear during the processing of complete dentures have an impact on the retention and stability of the denture [11,12]. The correct occlusion established after the final try-in may also be disturbed by the displacement of teeth in the course of the finalization of the complete dentures [10,13,14]. Inaccuracies in the occlusal scheme may result from clinical or technical judgment errors of the dentist or technical errors of the dental laboratory, and may also derive from deficiencies of the materials and techniques applied in denture construction [10,15,16]. Errors and inaccuracies should be corrected before the dentures are inserted into the patient’s mouth [10,17]. Corrections of occlusion constitute a time-consuming adjustment that often leads to disfigurement of the denture teeth anatomy [10,18].

The aim of this investigation was to analyze the accuracy of mandibular and maxillary digitally fabricated wax trial dentures and conventionally finalized complete dentures compared to a generated master surface tessellation language (STL)-dataset. The following null hypotheses were tested: (i) There is no difference regarding the accuracy between the master STL-dataset and the wax trial denture; (ii) There is no difference regarding the accuracy between the master STL-dataset and the finalized complete denture.

## 2. Materials and Methods

For this in vitro study, a previously generated dataset was used for the denture bases and the tooth sockets. The fabrication of the mandibular and maxillary dentures has been reported in a case report [1]. Data were converted into STL-format, and were saved as reference. Five dental maxillary and 5 mandibular stone casts (Resinrock; Whip Mix, Louisville, KY, USA) were prepared with the help of silicone molds of the definitive maxillary and mandibular casts of the case report. In order to ensure a complete setting of the plaster, the casts were stored 72 h.

Afterwards, 5 mandibular and 5 maxillary denture bases were milled with a 5-axis CAD/CAM-machine (Ceramill Motion 2; Amann Girrbach, Koblach, Austria) from a gingiva-colored wax blank (Ceramill D-Wax; Amann Girrbach) according to clinical practice. The wax dentures were cut out from the blank with a blade, as is customary in dental technical procedures. The bases of the wax dentures were checked before and after their removal from the blank. First, the wax trial dentures were placed on the casts, and the dorsal area was checked for gaps in order to ensure a correct fit of the wax bases on the casts. Then, the wax dentures were waxed onto the dental casts. The denture teeth (Pala Mondial 6/8; Kulzer, Hanau, Germany) were carefully waxed to the palatal/lingual regions of the sockets of the bases in the conventional way using a heated modelling instrument (PK-Thomas Color 2; Carl Martin, Solingen, Germany). Because of the space constraints, it was only possible to place the teeth in a certain direction. There was hardly any leeway, especially since the shape of the teeth was not symmetrical.

After 24 h, the wax trial dentures were digitized indirectly using an optical laboratory scanning device (strip light projection; Ceramill Map400; Amann Girrbach). For this purpose, the wax dentures were sprayed with scan powder (3-D Laserscanning-Entspiegelungsspray; Helling, Heidgraben, Germany) from a distance of 25 mm until all relevant areas were covered. This step was always carried out by the same operator. The scans were repeated until all the important points were scanned. During the matching procedure, the various scans were merged and reproduced as a complete denture. Scanning was not completed until all details were reproduced completely. The emphasis of the scans was placed on the teeth.

Subsequently, the wax trial dentures, including the casts, were positioned at the correct height and alignment in the lower halves of the denture flasks. For a better separation of the two flask halves after injecting, a smooth transition between the cast and the flask was prepared. The cast was carefully isolated (Ideal Isoliermittel; Klasse 4 dental, Augsburg, Germany) and injection channels were mounted. The denture teeth were covered with a plaster layer (pico-crema soft; picodent, Wipperfürth, Germany), the flask was closed and filled with plaster. After 45 min, the wax of the denture was boiled out. Then all plaster surfaces were pretreated twice with a plastic insulation (Aislar; Kulzer). The denture teeth were roughened using a coarse diamond cutter and conditioned with MMA solution (Palabond; Kulzer). Thereafter, the flask was closed and the MMA-based polymer (Palaxpress; Kulzer) was mixed according the manufacturer’s instructions. The flask was placed in the injection device (Palajet; Kulzer) and filled with polymer. After 5 min at 0.4 MPa, the flask was removed from the injection device and polymerized in a pressure pot (Palamat practice EL T; Kulzer) for 30 min at 55 °C and 0.2 MPa. Then, the flask was opened, and the plaster carefully removed with a plastic hammer. Subsequently, the complete denture was placed in an ultrasonic bath filled with a plaster solvent for 5 min. The injection channels and the denture edges were removed with a rough plastic cutter. The rest was left untreated. Subsequently, the complete dentures were scanned as described above and STL-datasets were generated. All investigations were carried out in air-conditioned rooms with a constant room temperature of 23 °C.

**Figure 1:**
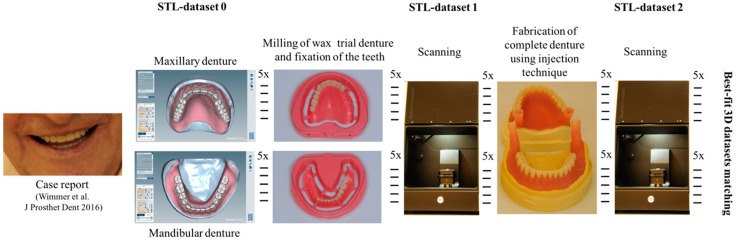
Study design including all processing steps.

The resulting STL-datasets were exported into the 3D software GOM Inspect (Version V8 Hotfix8; GOM, Braunschweig, Germany). The spatial differences between the dataset and the wax trial dentures or completed acrylic resin dentures were analyzed quantitatively. All datasets were reduced to the field of interest by elimination of all artifacts and not-relevant areas below the cast edge. In general, each of the 5 mandibular and 5 maxillary complete dentures was aligned with the STL- and the wax trial denture dataset. Alignment was performed based on a best-fit algorithm. To guarantee correct and reproducible alignment, a mounted plaster model served as reference. Thereafter, a 3D analysis of the spatial divergences in the *x*-, *y*- and *z*-axes was performed by the 3D software and visualized in a color-coded illustration. The mean positive and negative deviations between the datasets were calculated automatically. Figure 1 presents the study design, including all processing steps.

## 3. Results

The scale was set to the range of −1 mm to +1 mm. Areas where wax had been applied, such as on the teeth, were not considered in the evaluation. This also applies to the areas where the connectors were separated from the blank.

In direct comparison between maxillary wax trial dentures and complete dentures, complete dentures showed higher deviations from the STL-dataset than the wax trial dentures (Table 1). The deviations occurred both near the teeth and in the distal area of the denture bases. For the wax trial dentures, displacements in the positive direction were observed. The largest discrepancies were observed in wax denture No. 2 on the occlusal surfaces of the teeth (Table 1, No. 2 A–D). This denture also showed significant deviations around the molars in the fourth quadrant with values of approximately 0.40 mm. In addition, tooth 22 showed an inclination of 0.64 mm in the direction of the palate (Table 1, No. 2 D). A vertical increase of occlusion in the area of the posterior teeth was also observed for the remaining 4 wax dentures, which deviated up to approximately 0.40 mm (Table 1, No. 2 A–C). The denture bases showed maximum deviations in the range of 0.20 mm to 0.30 mm in the distal area. The complete dentures showed greater deviations in the distal areas of the denture bases, as well as on the occlusal surfaces of the teeth. The complete dentures No. 1–3 showed maximum deviations in the range of 0.8 mm and 0.9 mm (Table 1, No. 1–3). For complete denture No. 5, an occlusal accuracy of 0.67 mm was observed. In the distal areas of the denture bases, deviations of 0.65 mm to 0.80 mm were observed palatal and 0.45 mm to 0.65 mm in the area of the maxillary tuberosity (Table 1, No. 5 C,D).

Among the mandibular dentures, the largest deviations occurred in the distal areas both in the wax dentures and in the complete dentures. For the wax dentures, wax denture No. 1 showed maximum deviations of 0.63 mm in the positive direction for tooth 47 (Table 1, No. 1 A–D). In wax dentures No. 3–5, the molars showed a maximum vertical increase of occlusion between 0.40 and 0.50 mm (Table 1, No. 3–5). The premolars presented a deviation of about 0.30 mm. Around the retromolar triangle, the denture bases deviated by at least 0.40 mm from the desired situation. There were no deviations in the anterior tooth area. The complete dentures showed the highest deviations on the occlusal surfaces of the teeth. A positive displacement of the teeth was observed, especially in complete denture No. 3 (Table 1, No. 3 A–D). In the fourth quadrant, maximum values of about 1 mm occurred. In the complete dentures No. 1, 4 and 5, the largest deviation was observed for tooth 47. Here, maximum values of 0.95 mm (No. 1), 0.87 mm (No. 4) and 0.83 mm (No. 5) were obtained. The premolars also showed deviations with values over 0.50 mm (Table 1, No. 1, 4 and 5). The denture base area around the retromolar triangle showed maximum values of approximately 0.50 mm. The rest of the denture base was very close to the desired situation, except complete denture No. 3 (Table 1, No. 3 B).

## 4. Discussion

To facilitate the fabrication of complete dentures, a workflow for digital application was developed and implemented in conjunction with established CAD/CAM technology. The workflow considers the tooth arrangement, the milling of the wax trial bases, and the modification of the denture teeth so that they can be inserted into the tooth sockets of the bases with wax without additional grinding (Ceramill Full Denture Workflow; Amann Girrbach). In comparison to the conventional fabrication of complete dentures, the digital procedure provides several advantages; namely, a better detection and visualization of the morphology of the edentulous maxillary and mandibular arches, the facility to identify anatomic characteristics, and the possibility to identify and mark the midlines of the alveolar ridges automatically, for example. As a result of all of this, the implementation of digitally fabricated complete dentures establishes a reproducible procedure enabling predictable and accurate results as corroborated in the present study.

When comparing the accuracy of the wax trial dentures with the complete dentures, a clear difference with regard to the fit was observed. The mandibular and maxillary wax trial dentures already showed deviation from the STL-dataset. Yet, the deviations of the complete dentures even resulted in increased values. The observed inaccuracies of the complete dentures may have resulted from inaccuracies that were already present in milled wax trial dentures, and which had evolved throughout the processing, such as press flags in the area of the denture teeth. Moreover, it is not guaranteed that the teeth in the CAD software are identical to the real denture teeth. This can lead to deviations in matching the STL-dataset with the dataset of the scanned wax trial dentures. However, a more comprehensive investigation that preceded this present study determined and approved the reproducibility of digitally fabricated complete dentures.

Another step, such as the transfer of the wax into the acrylic resin complete denture, causes further inaccuracies. These inaccuracies during the transfer have already been observed in earlier studies [15]. Thus, it would be advantageous to make progress in the digital fabrication of dentures by directly milling in acrylic instead of wax milling disks. On the one hand, the unfavorable material properties of wax itself, which include warping and instability caused by temperature changes, constitute one plausible reason. On the other hand, the molding technique is widely known to have a higher degree of inaccuracy than the injection procedure [11]. In the present study, an injection procedure for the fabrication of complete dentures was used. This is the best rated method in terms of polymerization shrinkage [16]. In this investigation, the wax trial dentures were positioned and firmly embedded in plaster. In contrast, with regard to the casting process, it is possible for the teeth to shift in the elastic duplicating silicone. The shrinkage of PMMA-based materials can result in insufficient denture retention of complete dentures [12]. Also, partial damages of the plaster cast introduced during the cast out of the wax using a steam-jet air ejector might be further explanations for the inaccuracies of the acrylic resin complete dentures, as well as an irregular application of insulating agent and the heat development during the setting reaction of the plaster cast, which may have led to alterations of the embedded wax dentures.

Darvell et al. reported that denture retention is a dynamic issue, dependent on the control of the fluid flow between denture and oral mucosa—and hence the fluid’s viscosity and film thickness—with the timescale of the displacement loading having an effect on the assessment [12]. Surface tension forces at the periphery lead to increased retention, but a good base adaptation and border seal are the most important factors that must be achieved, if full advantage is to be taken of the saliva flow-related effects. In this study, a minimal increase in vertical occlusion was observed for the milled wax trial dentures. The transfer into the complete dentures using a PMMA-based material caused an additional and significantly higher increase in the occlusion. This suggests that the prostheses should be grinded-in, which leads to disfigurement of the denture teeth anatomy. Moreover, insufficient polishing of the grounded-in denture teeth can lead to a significantly higher abrasion rate. This reduces the survival rates of the complete denture. Inaccuracies within digitally fabricated complete dentures were also observed in another study, where rapid prototyping was used for the fabrication of the dentures [8]. In this study, maximum deviations of up to 0.88 mm occurred in the area of the denture teeth, and the authors suspect that the denture teeth did not fit correctly into the prefabricated tooth sockets. A further assumption for the positive deviations in the vertical direction may be the use of adhesive for the fixation of the teeth into the sockets. In the present study, the wax trial denture was milled, and the transfer into the complete denture was performed using the conventional method. For the bond between the denture teeth and the PMMA base, no additional adhesive system was needed, which could have increased the occlusion and impacted the accuracy negatively.

A further limitation of the study is the application of scan powder to enable a sufficient scan quality of the surface. This can lead to different layer thicknesses, and might have minimally influenced the results. To minimize the impact of scan powder, this step was always carried out by the same operator. This person applied a thin layer of powder from a distance of 25 cm until all relevant areas were covered, and proceeded according to the same protocol.

For prospective investigations, the following aspects should be considered: the effect of scan spray application on either wax or PMMA, the resulting possible impacts on the scans, and the robustness of the best-fit algorithm should be analyzed, as well as undertaking a comparison to 3D-printed complete dentures.

## 5. Conclusions

CAD/CAM-fabricated wax trial dentures showed less deviation from the STL-dataset than complete dentures. The deviations were observed in the area of the teeth and the distal area of the denture bases. These inaccuracies, resulting during the conventional transfer of the wax trial denture into the complete denture, depend on the behavior of the materials based on PMMA and the selected technique. In summary, both defined null hypotheses had to be rejected, because deviations from the master STL-dataset were observed in both—the wax trial dentures as well as the finalized complete dentures.

The clinical impact of this study demonstrates that the digital fabrication of denture bases made of wax enables a reproducible manufacturing process. Certainly, the manual positioning and fixation of denture teeth still result in minimal deviations, but the overall findings indicate a continuous improvement of reproducibility and accuracy for digitally fabricated dentures.

No matter, whether further developments of the digital manufacturing concentrate on subtractive or additive processes, the improvement of reproducibility and accuracy will benefit from clinical trials.

## Figures and Tables

**Table 1:** Matched STL-datasets about teeth.

	Mandibular Dentures		Maxillary Dentures	
Denture No.	STL vs. Wax Trial Denture	STL vs. Complete Denture	STL vs. Wax Trial Denture	STL vs. Complete Denture
	A	B	C	D
1	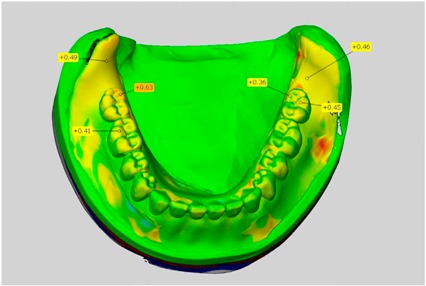	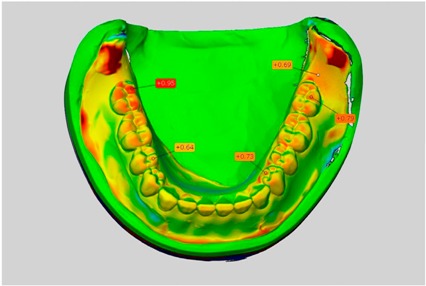	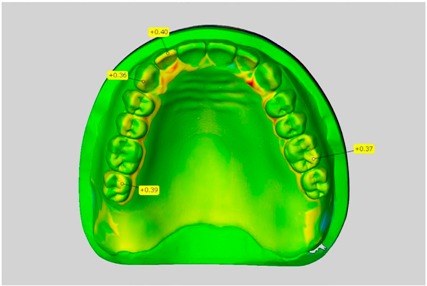	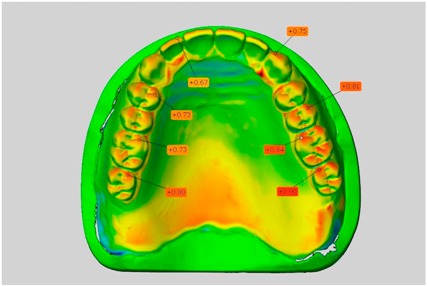
	A	B	C	D
2	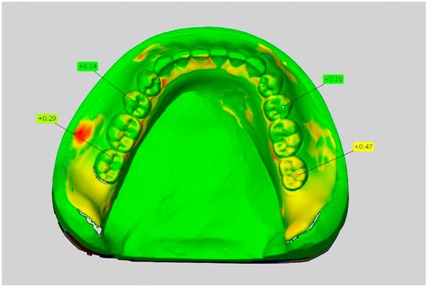	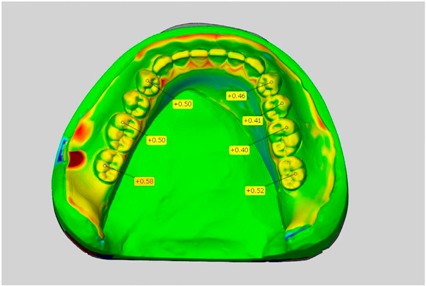	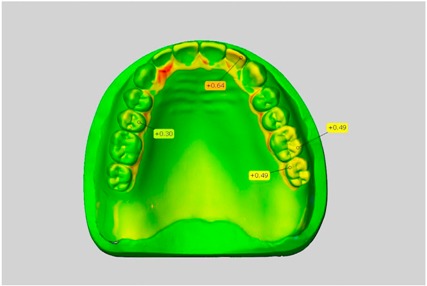	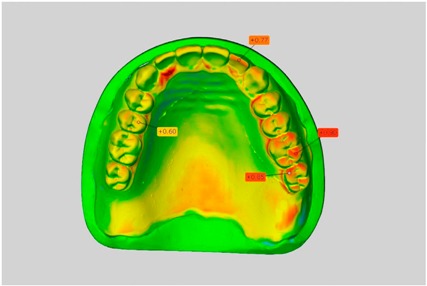
	A	B	C	D
3	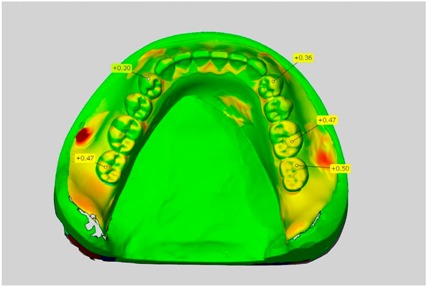	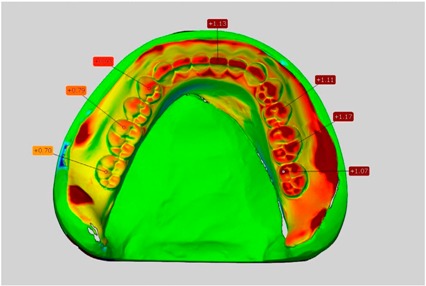	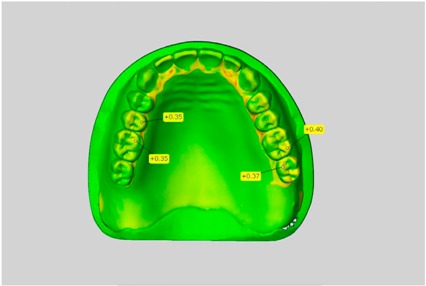	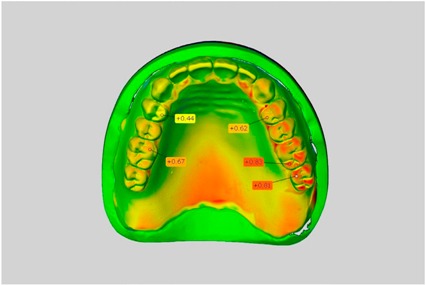
	A	B	C	D
4	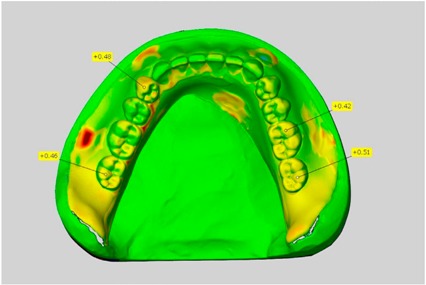	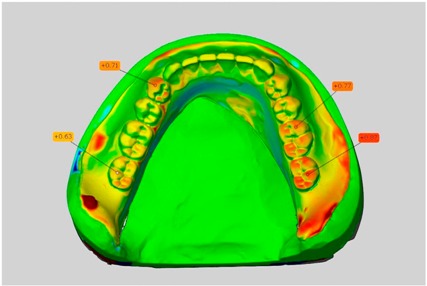	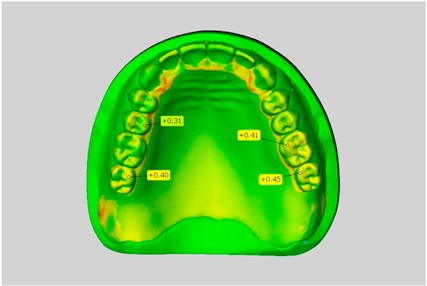	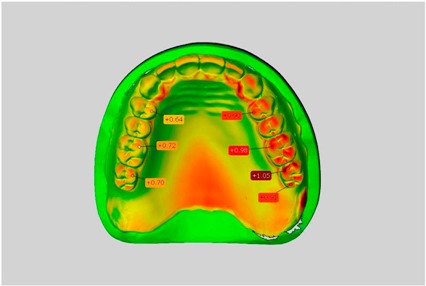
	A	B	C	D
5